# Stromal FAP Expression is Associated with MRI Visibility and Patient Survival in Prostate Cancer

**DOI:** 10.1158/2767-9764.CRC-21-0183

**Published:** 2022-03-21

**Authors:** Teijo Pellinen, Kevin Sandeman, Sami Blom, Riku Turkki, Annabrita Hemmes, Katja Välimäki, Juho Eineluoto, Anu Kenttämies, Stig Nordling, Olli Kallioniemi, Antti Rannikko, Tuomas Mirtti

**Affiliations:** 1 Institute for Molecular Medicine Finland (FIMM), Helsinki Institute of Life Science (HiLIFE), University of Helsinki, Helsinki, Finland.; 2 Department of Pathology, University of Helsinki and Helsinki University Hospital, Finland.; 3 Research Program in Systems Oncology, Faculty of Medicine, University of Helsinki, Helsinki, Finland.; 4 Science for Life Laboratory, Department of Oncology & Pathology, Karolinska Institutet, Stockholm, Sweden.; 5 Department of Urology, University of Helsinki and Helsinki University Hospital, Helsinki, Finland.; 6 Department of Radiology, University of Helsinki and Helsinki University Hospital, Helsinki, Finland.; 7 iCAN-Digital Precision Cancer Medicine Flagship, Helsinki, Finland.

## Abstract

Some clinically significant prostate cancers are missed by MRI. We asked whether the tumor stroma in surgically treated localized prostate cancer lesions positive or negative with MRI are different in their cellular and molecular properties, and whether the differences are reflected to the clinical course of the disease.

We profiled the stromal and immune cell composition of MRI-classified tumor lesions by applying multiplexed fluorescence IHC (mfIHC) and automated image analysis in a clinical cohort of 343 patients (cohort I). We compared stromal variables between MRI-visible lesions, invisible lesions, and benign tissue and assessed the predictive significance for biochemical recurrence (BCR) and disease-specific survival (DSS) using Cox regression and log-rank analysis. Subsequently, we carried out a prognostic validation of the identified biomarkers in a population-based cohort of 319 patients (cohort II).

MRI true-positive lesions are different from benign tissue and MRI false-negative lesions in their stromal composition. CD163^+^ cells (macrophages) and fibroblast activation protein (FAP)^+^ cells were more abundant in MRI true-positive than in MRI false-negative lesions or benign areas. In MRI true-visible lesions, a high proportion of stromal FAP^+^ cells was associated with PTEN status and increased immune infiltration (CD8^+^, CD163^+^), and predicted elevated risk for BCR. High FAP phenotype was confirmed to be a strong indicator of poor prognosis in two independent patient cohorts using also conventional IHC.

The molecular composition of the tumor stroma may determine whether early prostate lesions are detectable by MRI and associates with survival after surgical treatment.

**Significance:**

These findings may have a significant impact on clinical decision making as more radical treatments may be recommended for men with a combination of MRI-visible primary tumors and FAP^+^ tumor stroma.

## Introduction

A major challenge in the management of prostate cancer is to distinguish patients with clinically significant prostate cancer (csPCa, i.e., aggressive and potentially lethal prostate cancer) from patients with clinically insignificant prostate cancer, an indolent prostate cancer that does not affect the patient's life expectancy. Several biomarkers and tools have been developed to improve diagnostic accuracy and guide patient management (1). Still, overdiagnosing clinically insignificant prostate cancer and underdiagnosing csPCa is a major problem, and better biomarkers and stratification tools are urgently needed.

MRI is currently used regularly in prostate cancer diagnosis (2). It is typically reported in a structured manner using Prostate Imaging Reporting and Data System (PI-RADS; categories 0 to 5; ref. 3). PI-RADS has improved preoperative prediction of csPCa while also providing information on clinical staging (4–10). MRI is advantageous in targeting biopsies to index lesions (11). However, the false-negative MRI rate varies between 5% and 20%, depending on the cohort studied (12–14). Cellularity correlates with diffusion-weighted imaging (DWI)-derived apparent diffusion coefficients (ADC), PI-RAD values, and thus MRI visibility (PI-RADs 3–5), although the results are somewhat conflicting (15–17). A higher Gleason Grade and larger tumors are associated with higher PI-RADS categories, whereas smaller and multifocal tumors may mimic normal prostate tissue in MRI findings (13, 18, 19). Furthermore, growing evidence suggests that other histologic features, such as cancer versus stroma content or luminal area, contribute to variable MRI results (15, 20, 21). Notably, MRI true-positive prostate cancer more frequently contains tumor-associated desmoplastic stroma (22), suggesting that the tumor microenvironment (TME) architecture and composition may considerably influence MRI findings. However, differences in the molecular and cellular composition of the TME between MRI true-positive and MRI false-negative lesions and benign prostate tissue remain largely unexplored.

Using multiplexed fluorescence IHC (mfIHC) and digital image analysis, we recently showed that a higher number of fibroblasts and a lower number of smooth muscle cells in localized prostate cancer predicts poorer survival at radical prostatectomy (23). Here, we sought to investigate the association of stromal components to MRI visibility in a matched multifocal patient tissue cohort. Furthermore, we explored whether stromal signatures associated with MRI visibility in prostate cancer are reflected in patient survival.

## Materials and Methods

### Patient Cohorts and Tissue Microarrays

#### Cohort I (MRI-RALP Cohort)

The cohort consisted of 387 patients with preoperative multiparametric MRI and subsequent robot-assisted laparoscopic prostatectomy (RALP) as their primary treatment at the Department of Urology in Helsinki University Hospital (Helsinki, Finland) between January 2014 and September 2015. The design and generation of tissue microarrays (TMA) for cohort I has been previously described (24). After linking tumor location data, preoperative MRI data, and mfIHC data, 38 of the 387 patients were excluded due to insufficient matching data. Gleason Grade grouping was visually assessed from both whole hematoxylin and eosin (H&E) sections and TMA cores. After a thorough quality check by visual analysis of the H&E-stained TMAs and of the multiplex stained and digitally imaged TMAs, 6 more patients were excluded. Consequently, 343 patients with a total of 1,606 TMA cores were available (see REMARK diagram, Supplementary Fig. S1). The 343-patient TMA cohort was used for mfIHC quantifications and correlation analyses. Cohort I characteristics are shown in Supplementary Table S1. For biochemical recurrence (BCR)-free survival analyses, 25 patients were excluded due to a missing BCR status or due to follow-up time <3 weeks after RP (final *N* = 318). BCR was defined as the increase of PSA from undetectable level to 0.2 ng/mL after RP. For survival analyses, altogether the number of MRI true-positive lesions was 280, MRI false-negative lesions 147, and benign areas 251. Analyses were performed in a case-based manner by averaging replicate core results.

#### Cohort II (Helsinki RP Cohort)

Cohort II was a continuous, population-based collection of radical prostatectomies as formalin-fixed, paraffin-embedded (FFPE) samples obtained from 1983 to 1998 in the Department of Pathology at the Helsinki University Hospital (Helsinki, Finland). The generation of TMA is described in a previous publication (25). Shortly, FFPE blocks from each patient were punched from the following areas: two cores from the area containing the most dominant Gleason grade pattern, one core from the area containing the second most dominant Gleason pattern, and one core from an adjacent benign glandular area. Cancer cores from the same patient were averaged in image analyses. Disease-specific survival (DSS) was recorded as an endpoint with a median follow up of 16.5 years. Patients receiving any neoadjuvant therapy were excluded from the analyses. Cohort characteristics are shown in Supplementary Table S2. After matching clinical data and good quality representative tissue, 319 patients were included in the final analyses. FAP staining was successfully evaluated from 311 patients.

### Ethics Approval

Ethical approvals for the use of human tissue material and clinicopathologic data were obtained from Institutional Ethics Committee of Hospital District of Helsinki and Uusimaa (§70/16.5.2018; HUS/419/2018) and by the Finnish Institute for Health and Welfare (D:no THL 1231/5.05.00/2015 and D:no THL 490.5.05.00/2016) according to the national legislation. The use of retrospective archived tissue blocks was approved by the National Supervisory Authority for Welfare and Health (VALVIRA, D:no V/38176/2018). According to the national and European Union legislation on noninterventional medical research, the study was conducted without informed individual patient consents by permission of the Hospital District of Helsinki and Uusimaa (§105/21.12.2018; HUS/419/2018). The experiments conformed to the principles set out in the WMA Declaration of Helsinki and the Department of Health and Human Services Belmont Report.

### MRI

The preoperative prostate MRI scans were conducted with Philips Achieva 3.0T device. The protocol consisted of T2 (T2WI), diffusion-weighted imaging (DWI) including ADC mapping and dynamic contrast enhanced sequence. Details of the protocol by each sequence are presented in Supplementary Table S3. The imaging protocol was consistent with the PI-RADS recommendations. The MRIs were reported according to the PI-RADSv1 (26) with a structured form including number of lesions (up to 4), location and size (volume, max diameter) of each lesion, capsule contact length, extraprostatic extension (EPE), seminal vesicle invasion (SVI), and lymph node metastasis (LNM). Four radiologists performed the reading with an average of 200 prostate MRI cases per year per person.

### mfIHC

The original protocol for mfIHC, imaging, and analysis has been previously described (27). The second-round chromogenic staining was now replaced by a second-round fluorescence staining. The following primary antibodies and detection reagents were used for the first round staining: CD8 (1:1,000; Dako M7103) with TSA-488 detection (Life Technologies), FAP (1:500; Abcam AB207178) with TSA-555 detection, CD163 (1:200, Abcam AB188571) with anti-rabbit-AF647 detection (Thermo Fisher Scientific), PanEpi (consists of three antibodies: PanCk C-11, 1:150, Abcam AB7753; PanCK AE1/3, 1:100, Invitrogen MA5–13156; E-cadherin, Becton Dickinson, 610182) with anti-mouse-AF750 detection (Abcam, AB175738). Slides were costained with DAPI (Roche, 1.6 µg/mL) and mounted with ProLong Gold (Thermo Fisher Scientific). After whole-slide imaging (see imaging below), the coverslips were removed by soaking the slides in wash buffer at 4°C. Then the previous Alexa Fluor staining was bleached by soaking the slides in TBS buffer containing 25 mmol/L NaOH and 4.5% H_2_O_2_. The antibodies from the first-round staining were denatured by heating the slides in 10 mmol/L Tris/ 1 mmol/L EDTA pH 9 solution for 20 minutes at 99°C. The second-round staining consisted of alpha-smooth muscle actin (SMA) antibody staining (1:200, Abcam AB32575) with anti-rabbit-AF647 detection (Thermo Fisher Scientific) and costaining with DAPI.

### mfIHC Imaging

Five-channel fluorescence images were acquired using Metafer 5 scanning and imaging platform (MetaSystems) consisting of AxioImager.Z2 (Zeiss) microscope equipped with Zeiss Plan-Apochromat 20× objective (NA 0.8), CoolCube 2 m CCD camera (MetaSystems), PhotoFluor LM-75 (89 North) metal-halide light source, and Zeiss EPLAX VP232–2 power supply. DAPI, FITC, Cy3, Cy5, and Cy7 filters were used with the following exposure times: DAPI = 5.3 ms, FITC (CD8) = 7.7 ms, Cy3 (FAP) = 3.7 ms, Cy5 (CD163) = 80 ms, Cy7 (PanEpi) = 400 ms, second round Cy5 (SMA) = 32.2 ms. Nine field-of-views were acquired per each TMA spot, composed using VSlide (Metasystems), and the images were exported as one tiled image per spot as Lossless compressed TIFFs (95% resolution) for image analysis.

### mfIHC Image Analysis

Images from the second-round staining were registered (overlayed) with first round channel images using DAPI signals from the first-round and second-round images as before (27). The image analysis was carried out using a cell image analysis software (CellProfiler version 2.2.0). The pipeline is based on pixel classification and quantification and consisted of (i) tissue compartment area/count detection and (ii) marker positivity detection for cell fraction analysis. Tissue compartment areas and counts were normalized with total tissue area/count and thus were reported as fractions from total tissue area/count. Marker defined cell areas were normalized to total stroma area. In the detection of tissue compartments, tissue was defined by thresholding the sum of all channel pixels, and epithelium was detected as epithelial gland objects (glandularity) by thresholding PanEpi channel within tissue area and converting image mask to objects. Stroma was defined by subtracting the epithelial image mask from total tissue. Epithelial gland lumens were detected through filling and subtracting of epithelial objects/mask. Cellularity (cell count) was defined by using “IdentifyPrimaryObjects” module through adaptive Otsu's thresholding of DAPI channel. Marker positivities were defined by using adaptive Otsu's automatic thresholding.

### IHC, Imaging, and Image Analysis with Ilastik Machine Learning

We used PTEN and ETS transcription factor ERG IHC data from previous work (24). TMA slides were costained with anti-FAP (ab207178 rabbit 1:1,300 dilution) plus anti-SMA (M0851, mouse 1:500 dilution) using anti-rabbit (DPVR55HRP, Bright Vision) and anti-mouse (DPVM55AP, Bright Vision) secondary antibodies and the following substrates: Bright Vision, Bright DAB BS04–110 and Liquid Permanent Red K0640, Dako. The slides were washed for 1 minute in water after each reaction. Slides were then counterstained with hematoxylin (1:10 water dilution, 30 seconds) and after water washing and air drying, the slides were mounted (Pertex).

The stained slides were imaged with Pannoramic 250 Flash III (3DHISTEC Ltd) using 20× Zeiss Plan-Apochromat 20× objective (NA 0.8; 0.25 µm/pixel). Images were exported as whole-slide TIFFs and TMA spots were cropped with FIJI Roi1 1-Click tool.

We used Ilastik-1.3.3 machine learning software for FAP and SMA pixel detection using the pixel classification tool. Here, all the pixels were classified either to empty (no stain), FAP-positive, SMA positive, or other tissue (hematoxylin positive + tissue background). Simple segments were exported as TIFFs and classified pixels were counted using CellProfiler. FAP- and SMA-positive pixel counts were then normalized with total tissue pixel counts.

### Statistical Analysis

Normality of data was tested using Kolmogorov–Smirnov test. Student *t* test (paired) or Mann–Whitney *U* test was used to test differences between two normally distributed and nonnormally distributed continuous variables, respectively. Correlations between continuous variables or between continuous variables and categorical variables were calculated using Pearson correlation and Spearman *ρ* correlation coefficient function (paired, two-tailed), respectively. For survival analyses, we used Cox proportional hazard regression model and Kaplan–Meier plots with Wald test and log-rank, respectively. Proportional hazard assumption was tested for each variable using Schoenfeld test. AUROC (receiver operating characteristic (ROC) curve comparison using DeLong test for two correlated ROC curves, was used for studying whether a single-cell subtype may add prognostic value to the clinical variables and further, increase confidence in a prediction model. If multiple testing was performed, *P* values were controlled for using Bonferroni correction. *P* values <0.05 were considered significant. All statistical analyses were performed using IBM SPSS 26 (SPSS Inc.) or R Statistical Software v.3.3.2 (Foundation for Statistical Computing, Vienna, Austria). Data were plotted using R, SPSS 26, or Microsoft Excel.

### Data Availability

The data generated in this study are available upon request from the corresponding author.

## Results

### MRI False-Negative Lesions Resemble Benign Tissue More than MRI True-Positive Lesions in their Tissue Architecture

**FIGURE 1:**
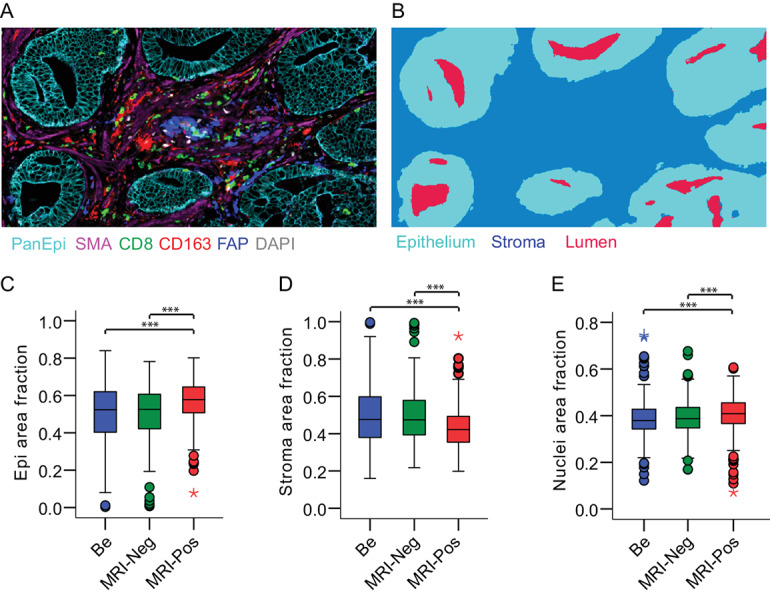
Tissue compartment differences in benign tissue areas, MRI false-negative cancer lesions, and MRI true-positive cancer lesions (*n* = 343 patients, TMA cores = 1606). **A,** mfIHC example image of tissue stained with indicated markers (PanEpi for epithelium, SMA for alpha smooth muscle actin, CD8 for cytotoxic T cells, CD163 for macrophages, FAP for fibroblast activation protein, and DAPI for nuclei. **B,** Example image of tissue segmentation for epithelium, stroma, and glandular lumen. Quantification of MRI-annotated tissue samples for epithelium area (**C**), stroma area (**D**), and nuclei area (**E**). Fractions are from total tissue area. Be, benign; MRI-Neg, MRI false-negative cancer lesions; MRI-Pos, MRI true-positive cancer lesions. Replicate TMA cores were averaged. Boxplot boxes, interquartile range (IQR); error lines, data points within 1.5-fold IQR; circles, data points within 3-fold IQR; stars, extreme data points. Pairwise nonparametric Mann–Whitney *U* test with asymptotic two-tailed significance is shown (***, *P* < 0.001).

To elucidate the architecture and tissue compartment differences between MRI false-negative, MRI true-positive, and benign lesions, we first quantified the total epithelial, stromal, and nuclear tissue areas by using mfIHC and computerized image analysis in patient cohort I (MRI-RALP cohort; Fig. 1). We observed that MRI false-negative lesions and benign tissue areas resembled each other with regards to tissue characteristic measures (Fig. 1C–E). MRI true-positive lesions had significantly higher epithelial and nuclear frequencies but lower stromal content than MRI false-negative lesions or benign tissue areas (*P* < 0.001; Fig. 1C–E). MRI true-positive lesions also had a significantly higher number of epithelial glands and gland lumens than MRI false-negative lesions or benign areas (Supplementary Fig. S2A and S2B). The only measured tissue characteristic that was similar in both types of cancer lesions but different in benign tissue was lumen area, which was significantly higher in benign tissue (median 9.7%) than in either MRI false-negative or MRI true-positive cancer lesions (7.6–8.4%; Supplementary Fig. S2C).

### Stromal Cell Composition Is Different between Benign Tissue, MRI False-Negative Lesions, and MRI True-Positive Lesions

**FIGURE 2:**
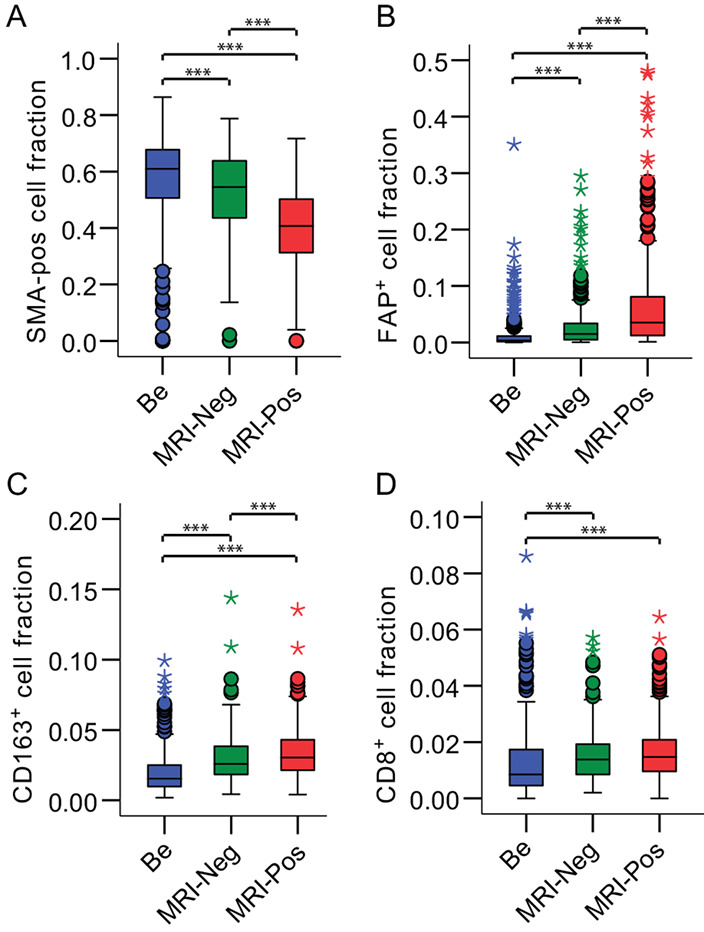
Stromal cell composition differences in RALP tissue samples with benign and MRI-annotated tissue areas. Marker-defined cell areas were normalized to the total stromal area. **A,** SMA-positive cell fraction. **B,** FAP-positive cell fraction. **C,** CD163-positive cell fraction. **D,** CD8-positive cell fraction. *N*(Be) = 309 patients, *N*(MRI-Neg) = 197 patients, *N*(MRI-Pos) = 302 patients. ***, *P* < 0.01. Boxplot boxes, interquartile range (IQR); error lines, data points within 1.5-fold IQR; circles, data points within 3-fold IQR; stars, extreme data points. Paired sample *t* test (normal distribution) or Mann–Whitney *U* test (nonnormal distribution) were used.

We analyzed the following stromal markers that reflect different stromal cell populations: CD8 (cytotoxic T cells), CD163 (cancer-associated macrophages), fibroblast activation protein (FAP, for cancer-associated fibroblasts), and alpha-smooth muscle actin (SMA, for smooth muscle cells). Stromal markers were measured within the stromal segment and their proportions were quantified in relation to the total stromal area. The SMA-positive cellular fraction was higher in the benign areas (median, 59.7%) than in either MRI false-negative lesions (median 55.9%) or in MRI true-positive lesions (median 41.3%; Fig. 2A). In contrast, the FAP-positive stromal cell proportion was lowest in benign areas (median 0.3%), increased in MRI false-negative lesions (median 1.4%), and was highest in MRI true-positive lesions (median 3.1%; Fig. 2B). The CD163-positive cell area behaved similarly to FAP positivity, as this was higher in MRI true-positive lesions (median 3.1%) than in either MRI false-negative lesions (median 2.5%) or in benign tissue areas (median 1.4%) (Fig. 2C). The CD8-positive cell fractions were similar between MRI true-positive (median 1.5%) and false-negative lesions (median 1.4%) but were significantly lower in benign areas (median 0.8%; Fig. 2D). These results indicate that prostate tumor stroma differs between benign tissue areas and cancer lesions (all markers), but importantly also between MRI true-positive and MRI false-negative lesions by CD163, FAP, and SMA positivity.

### FAP Correlates with CD8 and CD163 and Associates with PTEN Status and BCR

Stromal markers were reciprocally correlated using continuous data from all TMA cores in cohort I (*N* = 1,606; Supplementary Table S4). FAP and SMA had a mutual inverse correlation with each other (corr = −0.30, *P* < 0.01). FAP positively correlated with CD8 (corr = 0.44, *P* < 0.01) and with CD163 (corr = 0.43, *P* < 0.01), whereas SMA had a weak inverse correlation with CD8 (corr = −0.17, *P* < 0.01) and CD163 (corr = −0.21, *P* < 0.01). We explored whether the stromal markers were differentially expressed in MRI-classified lesions in patient subgroups defined by PTEN status, ERG status, Gleason grade grouping (GGG), and BCR. Interestingly, the highest correlation (inverse) was noted for stromal FAP positivity and tumor PTEN status (corr = −0.31, *P* < 0.01) in MRI true-positive lesions (Supplementary Table S5). This association was also significant in MRI false-negative lesions, albeit with a weaker correlation coefficient (corr = −0.15, *P* < 0.05; Supplementary Table S5). Patient cross-tabulation revealed that out of 47 patients with PTEN loss, 36 patients (76.6%) had higher than median FAP cell positivity (stromal FAP fraction >3.3%; Fisher exact test *P* < 0.001) in MRI true-positive lesions (Table 1). A higher SMA cell fraction was associated with positive PTEN (*P* = 0.025) and ERG (*P* = 0.001) expression status but also with lower GGG (*P* < 0.001; Table 1; Supplementary Table S5). Higher FAP cell fraction (*P* = 0.008) and lower SMA cell fraction (*P* = 0.001) were both associated with recurrent disease when measured in MRI true-positive lesions but not in MRI false-negative lesions (Table 1; Supplementary Table S5).

**TABLE 1:** Association analysis of FAP and SMA with PTEN, ERG, GGG, and BCR in cohort I TMA cores representing MRI-visible regions.

	FAP			SMA		
Variables	Low (*n* = 151)	High (*n* = 151)	*P* ^a^	Low (*n* = 151)	High (*n* = 151)	*P*
PTEN			**<0.001**			**0.025**
Neg (*n* = 47)	11(23%)	36(77%)		31 (66%)	16 (34%)	
Pos (*n* = 255)	140(55%)	115(45%)		120 (47%)	135 (53%)	
ERG			0.708			**0.001**
Neg (*n* = 210)	107 (51%)	103 (49%)		119 (57%)	91 (43%)	
Pos (*n* = 92)	44 (48%)	48 (52%)		32 (35%)	60 (65%)	
GGG			0.239			**<0.001**
1 (*n* = 6)	3 (50%)	3 (50%)		2 (33%)	4 (67%)	
2 (*n* = 112)	62 (55%)	50 (45%)		41 (37%)	71 (63%)	
3 (*n* = 143)	72 (50%)	71 (50%)		78 (55%)	65 (45%)	
4 (*n* = 10)	3 (30%)	7 (70%)		6 (60%)	4 (40%)	
5 (*n* = 31)	11 (34%)	20 (66%)		24 (77%)	7 (23%)	
BCR			**0.008**			**0.001**
No (*n* = 248)	133 (54%)	115 (46%)		113 (45%)	135 (55%)	
Yes (*n* = 32)	9 (28%)	23 (72%)		25 (78%)	7 (22%)	
NA (*n* = 42)						

^a^ *P* value (Fisher exact when applicable, *χ*^2^ when not). Significant *P* values are shown in bold.

### Stromal FAP and SMA in MRI True-Positive Lesions Predict BCR

We investigated whether the stromal mfIHC phenotypes measured in MRI true-positive and MRI false-negative lesions differentially associate with BCR in the MRI-RALP cohort I. We performed univariable Cox regression analysis using continuous image analysis values and compared these with clinical variables (Table 2). Because of multiple variables, we corrected the *P* values using Bonferroni correction. As expected with the clinical variables, the preoperative risk nomograms d'Amico and CAPRA predicted recurrence. None of the MRI variables reached significance in predicting BCR. Of the postoperative variables, tumor surface percentage, GGG, capsular invasion length, seminal vesicle invasion, and pathologic TNM stage (pTNM) predicted earlier recurrence. The analysis of continuous mfIHC variables in MRI true-positive cancer lesions showed that both a higher stromal fraction of FAP-positive cells and a lower stromal fraction of SMA-positive cells predicted earlier BCR. Interestingly, none of the mfIHC variables predicted BCR when measured from MRI false-negative lesions or benign tissue cores. However, as the number of patients in the MRI false-negative lesion group was low (*n* = 147) compared with that of benign (*n* = 251) or MRI true-positive lesions (*n* = 280), definitive outcome-related conclusions cannot be made for this group. Thus, subsequent survival analyses were performed using mfIHC measures from MRI true-positive lesions only.

**TABLE 2:** Univariable Cox regression analysis for clinicopathologic and mfIHC variables in cohort I.

Clinical variable	*P*	HR	*P* corr	mfIHC variable^a^	*P*	HR	*P* corr
Age	0.02	1.06	1.00	MRI-pos_Area_Nuclei	0.40	1.02	1.00
cT	0.01	1.60	0.50	MRI-pos_Area_Lumen	0.68	1.01	1.00
PSA	0.09	1.02	1.00	MRI-pos_Area_Epithelium	0.21	0.98	1.00
dAmico_risk	**0.00**	**2.73**	**0.01**	MRI-pos_Area_Stroma	0.21	1.02	1.00
CAPRA_risk	**0.00**	**2.93**	**0.00**	MRI-pos_Count_Lumen	0.46	1.00	1.00
MRIPROSTVOL	0.81	1.00	1.00	MRI-pos_CD163-pos cells	0.72	0.96	1.00
MRIFOCI	0.86	0.96	1.00	MRI-pos_CD8-pos cells	0.41	0.86	1.00
MRI1VOLUME	0.11	1.07	1.00	MRI-pos_FAP-pos cells	**0.00**	**1.04**	**0.01**
MRI1PIRADS	0.14	1.21	1.00	MRI-pos_SMA-pos cells	**0.00**	**0.96**	**0.01**
MRI1CAPSCONT	0.04	1.52	1.00	MRI-neg_Area_Nuclei	0.75	1.01	1.00
MRI1CAPSCONTMM	0.00	1.03	0.21	MRI-neg_Area_Lumen	0.61	0.98	1.00
MRIEPE	0.23	1.45	1.00	MRI-neg_Area_Epithelium	0.46	1.01	1.00
MRIEPE PI-RADS	0.13	1.12	1.00	MRI-neg_Area_Stroma	0.46	0.99	1.00
MRICLAS	0.20	0.12	1.00	MRI-neg_Count_Lumen	0.50	1.01	1.00
MRINCLAS	0.61	0.64	1.00	MRI-neg_CD163-pos cells	0.79	1.04	1.00
PROSTWEIGHT	0.95	1.00	1.00	MRI-neg_CD8-pos cells	0.81	1.06	1.00
FOCUSES	0.15	0.81	1.00	MRI-neg_FAP-pos cells	0.47	1.03	1.00
PERCSURFACE	**0.00**	**1.03**	**0.01**	MRI-neg_Sma-pos cells	0.39	0.99	1.00
GGG	**0.00**	**2.03**	**0.00**	Be_Area_Nuclei	0.08	1.04	1.00
POSMARG	0.00	1.05	0.26	Be_Area_Lumen	0.81	1.00	1.00
EPE	**0.00**	**1.08**	**0.00**	Be_Area_Epithelium	0.50	1.01	1.00
PN INV	0.11	2.70	1.00	Be_Area_Stroma	0.50	0.99	1.00
SVI	**0.00**	**3.37**	**0.03**	Be_Count_Lumen	0.26	1.03	1.00
pTNM	**0.00**	**1.25**	**0.00**	Be_CD163-pos cells	0.53	1.08	1.00
PTEN status	0.03	0.42	1.00	Be_CD8-pos cells	0.09	1.21	1.00
ERG status	0.17	0.54	1.00	Be_FAP-pos cells	0.94	1.00	1.00
				Be_Sma-pos cells	0.00	0.97	0.21

NOTE: Bolded values are significant with Bonferroni correction.

Abbreviations: Be, benign; EPE, extraprostatic extension; MM, millimeters; MRI-pos, MRI-positive lesion; MRI-neg, MRI-negative lesion; MRIPROSTVOL, MRI prostate volume; MRI1CAPSCONT, MRI capsular contact; MRICLAS, MRI classification; MRINCLAS, MRI N classification; *P* corr, Bonferroni-corrected *P*-value; PN INV, perineural invasion; pTNM, pathologic stage; SVI, seminal vesicle invasion.

^a^mfIHC variables as continuous values.

**FIGURE 3:**
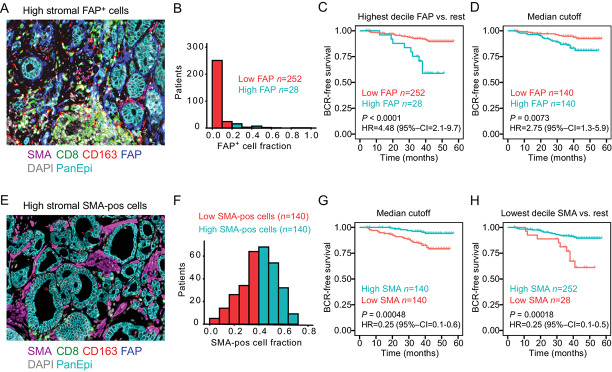
Stromal FAP and SMA in MRI true-positive lesions predict biochemical recurrence. **A,** Example case with high fraction of FAP^+^ stromal cells. **B,** Distribution of stromal FAP positivity (fraction from total stroma) (*n* = 280 patients). Red columns represent patients (90% of all the patients) with low FAP positivity (<20% fraction from stroma) and cyan columns represent patients with high FAP^+^ cell fraction (highest decile, 10% of patients). **C,** Kaplan–Meier plot for patients with low (red) and highest decile (cyan) FAP fraction. **D,** Kaplan–Meier plot for patients with low (red) and high (cyan) FAP fractions with median cut-off dichotomization. **E,** Example case showing high fraction of stromal SMA-positive cells. **F,** Distribution of stromal SMA positivity (fraction from total stroma; *n* = 280 patients). **G,** Kaplan–Meier plot of SMA-positive cell fraction with median dichotomization. **H,** Kaplan–Meier plot of SMA-positive cell fraction with dichotomization using lowest decile versus the rest of the patients. Kaplan–Meier plots show log-rank *P* values and HRs with 95% confidence intervals (95% CI) of univariable Cox regression survival analyses with the dichotomized values.

In Kaplan–Maier analysis guided by patient histogram distributions, dichotomization of patients with a cutoff at 20% FAP positivity or with a conservative median cutoff clearly stratified patients to poorer BCR-free survival with higher stromal FAP cell fraction (20% cutoff: HR, 4.48; 95% CI, 2.1–9.7; median cutoff: HR, 2.75; 95% CI, 1.3–5.9; Fig. 3A–D). In contrast, patients with a higher SMA-positive cell fraction in stroma had a more favorable outcome (median cutoff: HR, 0.25; 95% CI, 0.1–0.6; Fig. 3E–H). A stromal cell fraction positive for FAP but not for SMA (median cutoffs) remained prognostic also when adjusted for patient age, preoperative CAPRA risk, postoperative clinicopathologic variables (GGG, pTNM), as well as for SMA and PTEN status (Supplementary Fig. S3).

### FAP Is Validated as a Prognostic Marker Using a Different Staining Protocol and with Another Independent Patient Cohort

**FIGURE 4:**
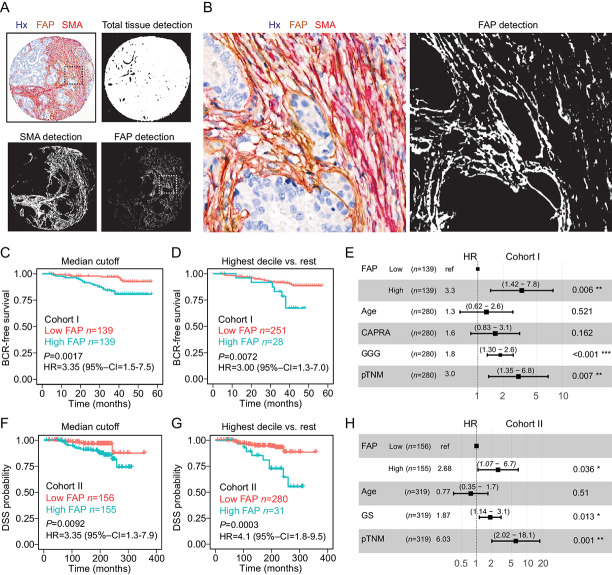
Validation of FAP as a prognostic factor. **A** and **B,** Double-antibody chromogenic staining for FAP and SMA (Hx, hematoxylin) with machine-learning detection of total tissue, FAP^+^ pixels, and SMA-positive pixels. **C** and **D,** Kaplan–Meier survival plots (BCR outcome) of patients with low and high FAP in MRI true-positive lesions determined by chromogenic IHC and machine learning (Cohort I; MRI-RALP cohort). **E,** Multivariable Cox regression analysis for median dichotomized FAP in MRI true-positive lesions (BCR outcome) in cohort I (chromogenic IHC). **, *P* <0.01; ***, *P* < 0.001. **F** and **G,** Kaplan–Meier survival plots for patients (cohort II) with low and high FAP in cancer lesions determined by chromogenic IHC and machine learning using the same algorithm as in cohort I (disease-specific survival, DSS outcome). **H,** Multivariable Cox regression analysis for median dichotomized chromogenic FAP in cancer lesions (DSS outcome) in cohort II (chromogenic IHC), *, *P* < 0.05; **, *P* < 0.01. 95% CI, 95% confidence interval.

Given that FAP associated with MRI visibility and disease progression independently of other clinical variables and SMA, we next investigated whether this could be quantitatively analyzed using a technique more easily applicable to the clinical setting. For this, we employed a double-antibody chromogenic IHC (FAP, brown; SMA, red) with hematoxylin counterstain (blue) and image analysis using a machine-learning approach (Fig. 4A and B; Ilastik machine learning, see Materials and Methods). Because of a missing epithelial costain and thus lack of epithelial–stromal segmentation, the FAP-positive area fraction was now measured from the total tissue area instead of the total stroma area. Analysis of the same MRI-RALP cohort I resulted in high concordance between fluorescent mfIHC and chromogenic IHC for the measured FAP-positive cell fraction (Pearson corr = 0.753; *P* < 0.001). The associations for high FAP and PTEN loss (Fisher exact test *P* < 0.001) and recurrent disease (Fisher exact test *P* = 0.004) were validated when FAP was measured using chromogenic IHC/machine-learning technology (Supplementary Table S6). Similarly, Kaplan–Maier survival analysis (Fig. 4C and D) and multivariable Cox regression analysis (Fig. 4E; Supplementary Fig. S4) demonstrated that high FAP tissue positivity is an independent factor predicting BCR in MRI true-positive lesions of the RALP samples. However, addition of FAP cell fraction as a variable to a risk model including patient age, CAPRA risk, GGG, and pTNM, did not further add did not further add prognostic power (Supplementary Table S7). We then applied the same chromogenic technique and the same machine-learning algorithm to another independent prostatectomy cohort (TMA cohort II), which is based on a continuous Finnish population patient series with DSS follow up and has been well characterized in earlier studies (Supplementary Table S2 for clinical characteristics; refs. 23, 25). A high FAP-positive tissue fraction, when dichotomized in the same manner using either median or highest decile cutoffs, also predicted DSS and was independent of patient age, Gleason score (GS), and pTNM class (Fig. 4F–H).

## Discussion

We explored the biology behind MRI visibility in this study. Our focus was in determining whether a specific stromal signature for MRI false-negative and true-positive lesions can be identified and if these correlate with clinically meaningful survival endpoints. This indeed seems to be the case, as the characteristics of the tumor stroma (fibroblasts and CD163-positive immune cells) are remarkably different between MRI false-negative and MRI true-positive lesions. We also report that stromal cells, particularly those that are positive for FAP, influence patient outcome in tumors, which are detectable by MRI (PI-RAD). Taken together, these results suggest a link between prediagnostic clinical imaging (MRI) and tissue biomarker status.

The diagnosis of prostate cancer currently relies on histologic confirmation by needle biopsies. The traditional diagnostic pathway that relies on PSA and systematic biopsies unfortunately leads to significant overdiagnosis of clinically insignificant prostate cancers. Furthermore, many clinically significant cancers are left undiagnosed. Recent randomized trials have highlighted the value of a contemporary, MRI-based diagnostic pathway. MRI significantly reduces overdiagnosis and improves detection of csPCa. However, approximately 5%–20% of csPCas are not visible in MRI (28). Thus, there is an unmet clinical need to evaluate the biological characteristics of MRI false-negative lesions and MRI true-positive lesions at the tissue level and to investigate whether any associated signal reflects a favorable or unfavorable clinical course.

The biological and molecular differences of MRI true-positive and MRI false-negative lesions have not been investigated in detail. Consistent with earlier reports on histologic characteristics (15, 20, 21), we found higher cellularity, glandularity, higher epithelial area, and lower lumen area in MRI true-positive tumor lesions than in MRI false-negative lesions or in benign tissue areas. Our study is in line with the suggestion that MRI-invisible tumors resemble normal prostate tissue more (29). Earlier work suggested that MRI true-positive tumors always contained desmoplastic stroma, whereas only 33.3% of MRI false-negative tumors had desmoplastic appearance (22). In desmoplasia or reactive stroma of prostate, elevated numbers of stromal fibroblasts and inflammatory cells mimic the repair process of wounding (30–32). This has been shown to associate with PTEN loss and cancer initiation and progression (33, 34). We found a remarkably higher proportion of CD163^+^ immune cells and FAP-positive fibroblasts in MRI true-positive tumor lesions compared with either MRI false-negative tumor lesions or benign tissue. Importantly, we also observed that FAP positivity significantly correlated with CD8 and CD163, but also with PTEN loss. Our results imply that stromal cellular composition contributes to MRI visibility and disease progression, and these findings together with earlier reports suggest that these could be linked with the presence of desmoplasia or reactive stroma. As FAP was prognostic only in patients with tumors classified as MRI positive, but not MRI negative, the findings suggest that FAP could be a relevant biomarker of prostate cancer progression, especially when combined with MRI diagnostics.

In this study, we also investigated whether high FAP and low SMA are independent predictors of BCR in MRI-positive tissue. Stromal FAP predicted BCR independently when adjusted for preoperative risk factors (CAPRA risk nomogram) and postoperative factors (GGG, pTNM), and was also independent of SMA and PTEN status. The predictive value of FAP was further confirmed in an independent cohort of surgical patients with a long follow-up time of >15 years and with a hard endpoint of disease-specific mortality. These results warrant for further prospective studies to investigate whether FAP could be used as a biomarker with an additional value to the current clinical risk factors both in preoperative and postoperative settings.

In conclusion, this study links specific primary tumor stroma biology with preoperative MRI visibility and prostate cancer progression to a metastatic disease. Importantly, these findings may have a significant impact on clinical decision making regarding systematic prostate biopsy versus follow up in men with negative MRI, whereas a more intensive follow-up and more radical treatments may be recommended for men with MRI-positive lesions and high stromal FAP content.

## Supplementary Material

Supplementary Tables S1-S7, Figures S1-S4 Supplementary Tables S1-S7. Supplementary Figures S1-S4.
