# Supplementary material for: Stromal FAP Expression is Associated with MRI Visibility and Patient Survival in Prostate Cancer
Source: Cancer Res Commun. 2022 Mar 21;2(3):172–81. doi: 10.1158/2767-9764.CRC-21-0183 (PMC9980917; doi:10.1158/2767-9764.CRC-21-0183)
Supplement: Supplementary Tables S1-S7, Figures S1-S4 — Supplementary Tables S1-S7. Supplementary Figures S1-S4. [file crc-21-0183-s01.docx]

**Supplementary Data to**

**Stromal FAP expression is associated with MRI visibility and patient survival in prostate cancer**

Teijo Pellinen^1,$,#^, Kevin. Sandeman^2,3,$^, Sami Blom^1,*^, Riku Turkki^1,4,**^, Annabrita Hemmes^1^, Katja Välimäki^1^, Juho Eineluoto^3,5^, Anu Kenttämies^6^, Stig Nordling^2^, Olli Kallioniemi^1,4^, Antti Rannikko^3,5,7^, Tuomas Mirtti^2,3,7^

^1^Institute for Molecular Medicine Finland (FIMM), Helsinki Institute of Life Science (HiLIFE), University of Helsinki, Helsinki, Finland

^2^Department of Pathology, University of Helsinki and Helsinki University Hospital, Finland.

^3^Research Program in Systems Oncology, Faculty of Medicine, University of Helsinki, Finland.

^4^Science for Life Laboratory, Department of Oncology & Pathology, Karolinska Institutet, Stockholm, Sweden

^5^Department of Urology, University of Helsinki and Helsinki University Hospital, Finland.

^6^Department of Radiology, University of Helsinki and Helsinki University Hospital, Finland.

^7^iCAN-Digital Precision Cancer Medicine Flagship, Helsinki, Finland.

^$^Shared first author

*Affiliation at the time the work was conducted; now affiliated with Aiforia Technologies Oy, Helsinki, Finland.

**Affiliation at the time the work was conducted; now affiliated with AstraZeneca, Gothenburg, Sweden.

^#^Corresponding author: Dr Teijo Pellinen, Institute for Molecular Medicine Finland (FIMM), Helsinki Institute of Life Science (HiLIFE), University of Helsinki, Tukholmankatu 8, 00290, Helsinki, Finland. Tel. +358503005688. Email: teijo.pellinen@helsinki.fi

**Supplementary Tables and Figures**

**Table S1. Patient clinical characteristics, cohort I (MRI-RALP), *N*=343.**

| **Pre-operative clinical data** | |
| --- | --- |
| Age (years) |  |
| Median | 64.8 |
| Range (min-max) | 43–79 |
| cT |  |
| 1 | 223 |
| 2 | 65 |
| 3–4 | 55 |
| PSA (ng/ml) |  |
| Median | 8.9 |
| Range (min-max) | 1.23–84 |
| d'Amico risk |  |
| 1 | 39 |
| 2 | 181 |
| 3 | 122 |
| CAPRA risk |  |
| 1 | 45 |
| 2 | 174 |
| 3 | 90 |
| **Pre-operative MRI data** | |
| MRI prostate volume | |
| Median | 35 |
| Range (min-max) | 12–125 |
| MRI foci (*N*) |  |
| 0 | 26 |
| 1 | 203 |
| 2 | 84 |
| 3 | 29 |
| 4 | 1 |
| MRI PI-RADS |  |
| 0 | 26 |
| 1 | 2 |
| 2 | 8 |
| 3 | 24 |
| 4 | 37 |
| 5 | 245 |
| MRI capsular contact | |
| 0 (no) | 111 |
| 1 (maybe) | 163 |
| 2 (yes) | 69 |
| MRI capsular contact | |
| Median (mm) | 12 |
| Range (mm) | 0–76 |
| MRI EPE |  |
| no | 221 |
| yes | 122 |
| MRI EPE PI-RADS |  |
| 0 | 174 |
| 1 | 41 |
| 2 | 0 |
| 3 | 17 |
| 4 | 59 |
| 5 | 52 |
| MRI T classification |  |
| 0 | 31 |
| 1c | 4 |
| 2 | 27 |
| 2a | 87 |
| 2b | 4 |
| 2c | 56 |
| 3 | 16 |
| 3a | 72 |
| 3b | 31 |
| 4 | 15 |
| MRI N classification |  |
| 0 | 336 |
| 1 | 6 |
| 2 | 1 |
| **Post-operative clinical data** | |
| Prostate weight |  |
| Median (g) | 48 |
| Range (g) | 22–119 |
| Tumor Foci (n) | 342 |
| 1 | 101 |
| 2 | 120 |
| 3 | 75 |
| 4 | 36 |
| 5 | 6 |
| 6 | 2 |
| 7 | 1 |
| 8 | 1 |
| Cancer proportion (%) |  |
| Median | 15 |
| Range (min-max) | 3–100 |
| GGG | 343 |
| 1 | 14 |
| 2 | 135 |
| 3 | 152 |
| 4 | 10 |
| 5 | 32 |
| Positive margin |  |
| no | 249 |
| yes | 92 |
| EPE |  |
| no | 215 |
| yes | 115 |
| If yes. median (mm) | 6 |
| PNI |  |
| no | 40 |
| yes | 303 |
| SVI |  |
| no | 301 |
| yes | 42 |
| pTNM |  |
| 2 | 208 |
| 3–4 | 135 |

cT, clinical tumor staging; PI-RADS, Prostate Imaging Reporting and Data System; MRI foci (*N*), number of tumor foci with MRI data; EPE, extra prostatic extension; GGG, Gleason Grade Group; PNI, perineural invasion; SVI, seminal vesicle invasion; pTNM, pathological stage.

**Table S2. Patient clinical characteristics, cohort II (RP cohort); *N*=319.**

| Follow-up time after surgery (median years) | 16.5 |
| --- | --- |
| Death from any cause (*N*) | 127 |
| Death from PCa (*N*) | 26 |
| Biochemical recurrence (*N*) | N/A* |
| Age (years) |  |
| Median | 64 |
| Range (min-max) | 45–76 |
| RP cancer proportion (%) |  |
| Median | 15 |
| Range (min-max) | 0–90 |
| RP Gleason score |  |
| 5 | 11 |
| 6 | 74 |
| 7 | 186 |
| 8 | 37 |
| 9 | 10 |
| pT |  |
| ≤2 | 184 |
| ≥3 | 127 |
|  |  |

*This series was collected partly prior to routine PSA follow-up

**Table S3. MRI protocol details.**

| Sequence | Slice thickness (mm) | Resolution (mm) | b-values | Temporal   resolution (s) | Acquisition time (s) | Flip Angle |
| --- | --- | --- | --- | --- | --- | --- |
| T2 | 3 | 0,6 x 0,7 |  |  |  |  |
| DWI^1^ for calculating ADC^2^ | 3 |  | 0, 100, 800 |  |  |  |
| DWI for detection | 3 |  | 2000 |  |  |  |
| DCE^3^ | 4 |  |  | 8 | 180 | 10 |

^1^Diffusion-weighted imaging, ^2^apparent diffusion coefficient, ^3^Dynamic contrast-enhanced (Dotarem®, 0.2 ml/kg, 2 ml/s).

**Table S4.** **TMA** **spot-level correlations of stromal mfIHC variables (Spearman rho) in cohort I**.

| Variable | CD163 cells | CD8 cells | FAP cells | SMA cells |
| --- | --- | --- | --- | --- |
| CD163-pos cells | 1 | 0.51** | 0.43** | -0.21** |
| CD8-pos cells | 0.51** | 1 | 0.44** | -0.17** |
| FAP-pos cells | 0.43** | 0.44** | 1 | -0.30** |
| SMA-pos cells | -0.21** | -0.17** | -0.30** | 1 |

***P*-value<0.01, two tailed, *N*=1606 TMA cores.

**Table S5. Patient-level correlations of mfIHC variables with genomic alterations (PTEN status, ERG status), Gleason grade grouping (GGG), and BCR status (Spearman rho) in cohort I.**

| Variable | PTEN_MRI- | PTEN_MRI+ | ERG_MRI- | ERG_MRI+ | GGG | BCR status |
| --- | --- | --- | --- | --- | --- | --- |
| CD163_Be | 0.08 | 0.01 | 0.00 | -0.05 | 0.02 | 0.05 |
| CD163_MRI- | -0.02 | 0.06 | 0.14* | 0.05 | -0.02 | 0.04 |
| CD163_MRI+ | 0.11 | -0.16** | 0.01 | 0.19** | 0.07 | 0.00 |
| CD8_Be | 0.07 | -0.11 | 0.01 | -0.02 | 0.06 | 0.09 |
| CD8_MRI- | 0.01 | -0.06 | 0.05 | 0.03 | -0.03 | 0.06 |
| CD8_MRI+ | 0.09 | -0.10 | 0.07 | 0.08 | -0.21* | -0.10 |
| FAP_Be | 0.03 | -0.10 | 0.02 | -0.03 | -0.02 | 0.07 |
| FAP_MRI- | -0.15* | -0.04 | 0.09 | 0.00 | -0.09 | 0.00 |
| FAP_MRI+ | 0.20* | -0.31** | -0.02 | 0.06 | 0.14* | 0.14* |
| SMA_Be | -0.15 | -0.04 | 0.03 | 0.02 | -0.06 | -0.15* |
| SMA_MRI- | -0.05 | 0.00 | 0.09 | 0.03 | -0.09 | -0.08 |
| SMA_MRI+ | -0.08 | 0.16** | 0.20* | 0.15** | -0.30** | -0.24** |

**P*-value<0.05, two tailed, ***P*-value<0.01, two tailed, *N*(Be)=272, *N*(MRI-)=197, *N*(MRI+)=302.

**Table S6. Crosstab association analysis of FAP and SMA with PTEN, ERG, GGG, and BCR status in cohort I TMA cores representing MRI-visible regions (chromogenic staining).**

|  | **FAP** | | | **SMA** | | |
| --- | --- | --- | --- | --- | --- | --- |
| **Variable** | **Low (*N*=139)** | **High (*N*=140)** | ***P*** | **Low (*N*=151)** | **High (*N*=151)** | ***P*** |
| **PTEN** |  |  | **<0.001** |  |  | 0.424 |
| Neg (n=42) | 8(19%) | 34(81%) |  | 22 (52%) | 20 (48%) |  |
| Pos (n=237) | 131(55%) | 106(45%) |  | 139 (50%) | 140 (50%) |  |
| **ERG** |  |  | 0.148 |  |  | **0.026** |
| Neg (n=210) | 104 (53%) | 47 (49%) |  | 106 (54%) | 91 (46%) |  |
| Pos (n=92) | 35 (43%) | 47 (57%) |  | 33 (40%) | 49 (60%) |  |
| **GGG** |  |  | 0.15 |  |  | **0.004** |
| 1 (n=6) | 5 (83%) | 1 (17%) |  | 5 (83%) | 1 (17%) |  |
| 2 (n=112) | 52 (51%) | 50 (49%) |  | 38 (37%) | 64 (63%) |  |
| 3 (n=143) | 69 (52%) | 64 (48%) |  | 77 (55%) | 56 (45%) |  |
| 4 (n=10) | 3 (30%) | 7 (70%) |  | 7 (60%) | 3 (40%) |  |
| 5 (n=31) | 10 (36%) | 18 (64%) |  | 12 (77%) | 16 (23%) |  |
| **BCR** |  |  | **0.004** |  |  | 0.459 |
| No (n=248) | 131 (53%) | 116 (47%) |  | 121 (49%) | 126 (51%) |  |
| Yes (n=32) | 8 (25%) | 24 (75%) |  | 18 (56%) | 14 (44%) |  |
| na (n=42) |  |  |  |  |  |  |

^1^*P*-value (Fisher’s exact when applicable, chi-square when not). Significant *P*-values are shown in bold.

**Table S7. AUROC analysis for risk prediction models.**

| **AUROC risk model** | **Variables** | **AUC of roc** | ***P*** |
| --- | --- | --- | --- |
| 1 | Age, CAPRA, GGG, pTNM | 0.7806 |  |
| 2 | FAP, Age, CAPRA, GGG, pTNM | 0.7915 | 0.6141 |

^1^*P, P*-value (DeLong’s test). AUROC, area under receiver operating characteristic (ROC) curve.

**
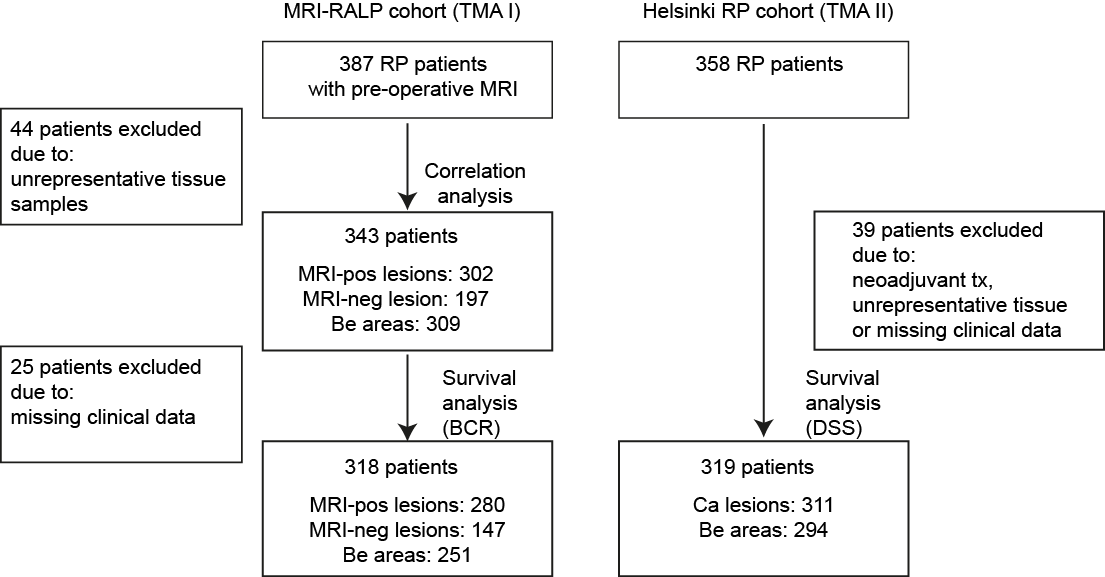
**

**Figure S1. REMARK diagram.** Study inclusion and exclusion criteria for prostate cancer patient samples in the MRI-RALP (cohort I) and RP (cohort II) cohorts. MRI, magnetic resonance imaging; RALP, robotic assisted laparoscopy prostatectomy; RP, radical prostatectomy; MRI-pos, MRI true-positive; MRI-neg, MRI false-negative; Be, benign; Ca, cancer; BCR, biochemical recurrence; DSS, disease-specific survival; tx=therapy.


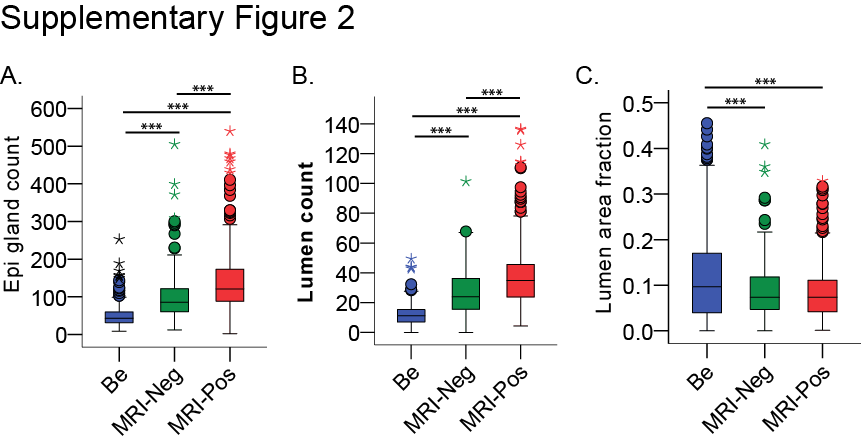


**Figure S2.** **Tissue compartment differences in benign tissue areas, MRI false-negative cancer lesions, and MRI true-positive cancer lesions.** **A.** Epithelial (Epi) gland count in each patient (*N*=343) as normalized by total tissue TMA core area and averaged by replicates. **B.** Lumen count in each patient TMA core as normalized by total tissue TMA core area and averaged by replicates. **C.** Lumen area fraction from total tissue area in a TMA core for each patient average of replicates. Pairwise nonparametric Mann-Whitney with asymptotic 2-tailed significances are shown (****P*<0.001).


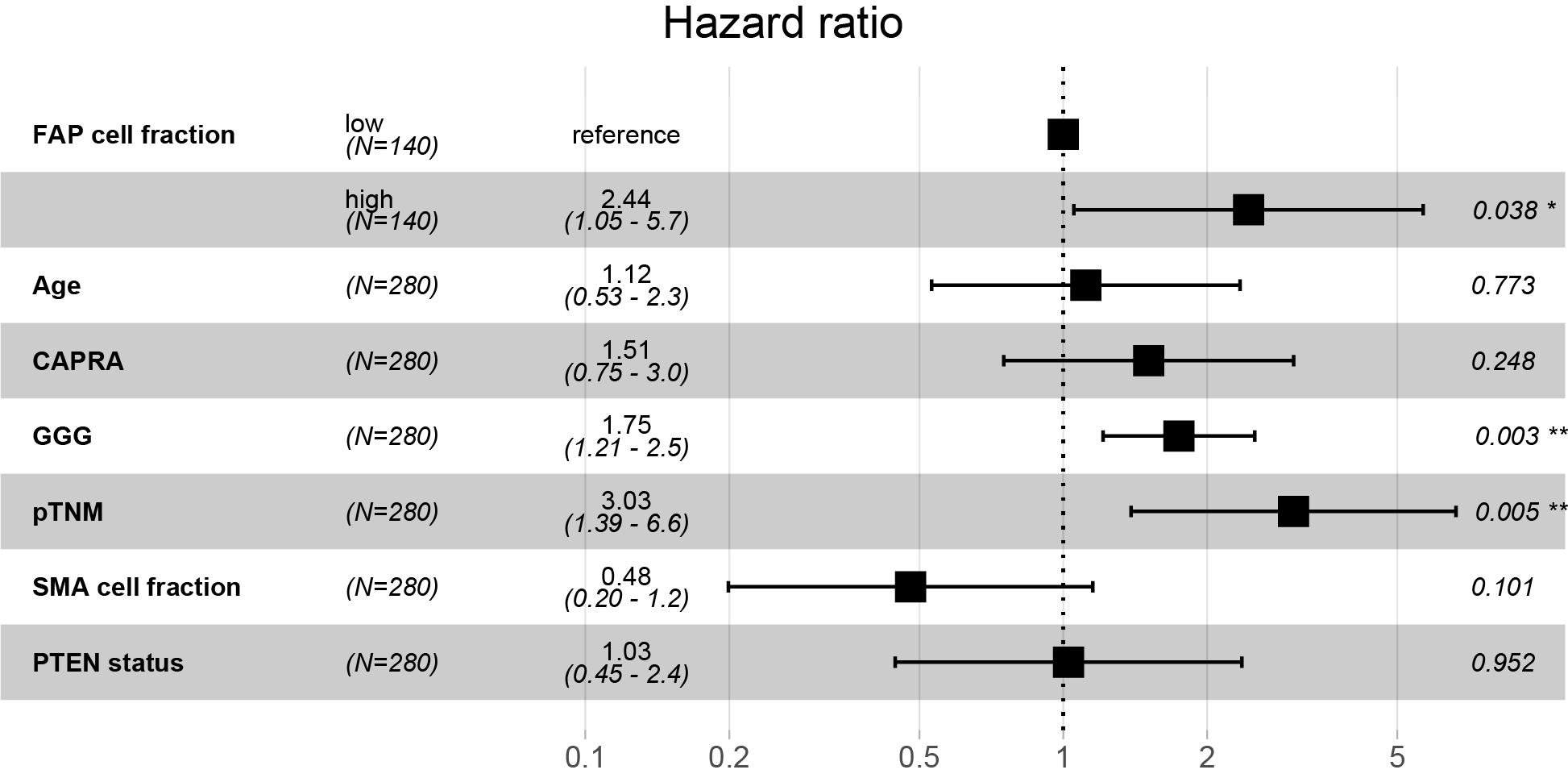


**Figure S3. Stromal FAP positive cell fraction is an independent predictor of BCR in true-positive MRI lesions in cohort I (Multiplex mfIHC measurements).** Multivariable Cox regression survival analysis with median dichotomized FAP and SMA, as measured using mfIHC in stromal cells. FAP and SMA positive stromal cell fractions were adjusted with Age (median cut-off 65 years), pre-operative CAPRA risk (scores 1 to 3), post-operative Gleason grade group (GGG scores 1 to 5), pathological TNM stage (pTNM; 2 vs. 3-4), as well as with stromal SMA cell fraction (median cut-off) and tumor PTEN status (null vs. positive). **P*-value<0.05, ***P*-value<0.01 (two tailed).


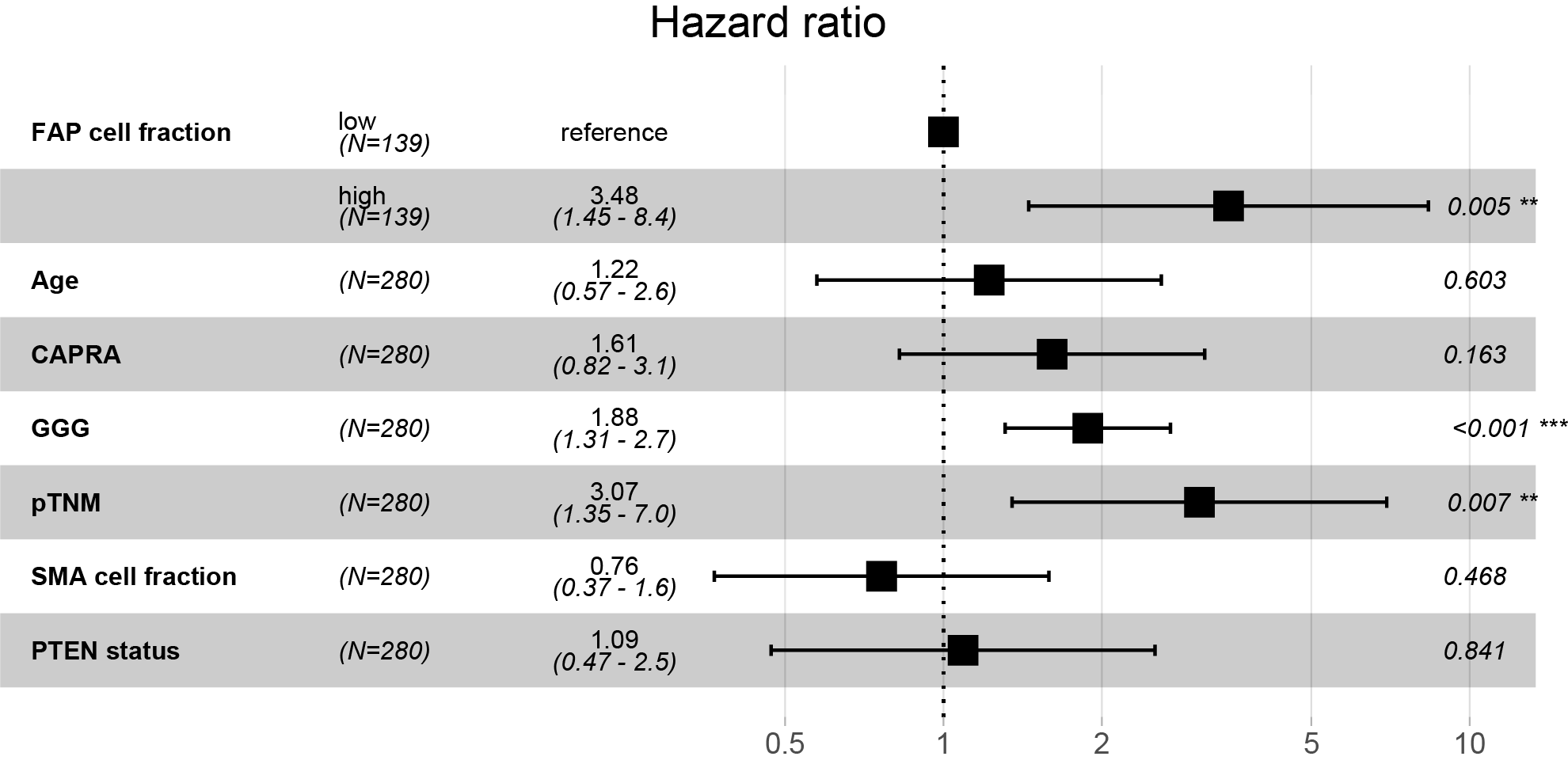


**Figure S4. FAP positive cell fraction is an independent predictor of BCR in true-positive MRI lesions in cohort I (chromogenic IHC + machine learning measurements).** Multivariable Cox regression survival analysis with median dichotomized FAP and SMA, as measured using conventional IHC and machine learning in cells of total tissue. FAP and SMA positive stromal cell fractions were adjusted with Age (median cut-off 65 years), pre-operative CAPRA risk (scores 1 to 3), as well as post-operative Gleason grade group (GGG scores 1 to 5), pathological TNM stage (pTNM; 2 vs. 3-4), as well as with stromal SMA cell fraction (median cut-off) and tumor PTEN status (null vs. positive). **P*-value<0.05, ***P*-value<0.01 (two tailed).
